# Global and localized network characteristics of the resting brain predict and adapt to foreign language learning in older adults

**DOI:** 10.1038/s41598-022-07629-y

**Published:** 2022-03-07

**Authors:** Maria Kliesch, Robert Becker, Alexis Hervais-Adelman

**Affiliations:** 1 Zurich Center for Linguistics, University of Zurich, Andreasstrasse 15, 8050 Zürich, Switzerland; 2 Chair of Romance Linguistics, Institute of Romance Studies, University of Zurich, Zürich, Switzerland; 3 Neurolinguistics, Department of Psychology, University of Zurich, Zürich, Switzerland; 4 Neuroscience Center Zurich, University of Zurich and Eidgenössische Technische Hochschule Zurich, Zürich, Switzerland

**Keywords:** Language, Neural ageing

## Abstract

Resting brain (rs) activity has been shown to be a reliable predictor of the level of foreign language (L2) proficiency younger adults can achieve in a given time-period. Since rs properties change over the lifespan, we investigated whether L2 attainment in older adults (aged 64–74 years) is also predicted by individual differences in rs activity, and to what extent rs activity itself changes as a function of L2 proficiency. To assess how neuronal assemblies communicate at specific frequencies to facilitate L2 development, we examined localized and global measures (Minimum Spanning Trees) of connectivity. Results showed that central organization within the beta band (~ 13–29.5 Hz) predicted measures of L2 complexity, fluency and accuracy, with the latter additionally predicted by a left-lateralized centro-parietal beta network. In contrast, reduced connectivity in a right-lateralized alpha (~ 7.5–12.5 Hz) network predicted development of L2 complexity. As accuracy improved, so did central organization in beta, whereas fluency improvements were reflected in localized changes within an interhemispheric beta network. Our findings highlight the importance of global and localized network efficiency and the role of beta oscillations for L2 learning and suggest plasticity even in the ageing brain. We interpret the findings against the background of networks identified in socio-cognitive processes.

## Introduction

It has long been believed that an individual’s success in mastering a new language depends largely on their age. This assumption was based on the observation that children seem to acquire their first language (L1) fast and effortlessly, whereas adults often require years of instruction to reach a comparable proficiency. More recent research, however, has repeatedly shown that in adults, the ability to learn a new language (L2) differs significantly between individuals and varies as a function of cognition, motivation and other background variables^1–3^. Importantly, the typical modern L2 learner is not necessarily a younger adult; older adults aged 60 + exhibit a similarly high interest in learning a new language as 30 + year olds^4^. While aging is associated with a stabilization of affective variability and motivation^5,6^, it is also characterized by a progressive decline in cognitive performance, especially in the domains of attention, memory and executive function^7^. These age-related changes also manifest on the neurophysiological level, such that older adults commonly show a slowing of the brain’s most dominant rhythm, the so-called alpha rhythm (8–13 Hz), and an increase in the slower delta (2–4 Hz) and theta (4–8 Hz) rhythms^8^. While research on the relationship between cognitive ability and L2 learning has gained momentum in recent years^3,9,10^, neural predictors of L2 development in older adults remain largely unknown^11^.

The ease with which a new language is learned in adulthood has been associated with a constellation of cognitive abilities^12^, including—but not limited to—working memory^1,3,10^, fluency in the L1^3,13,14^, attention^3,15,16^ and inhibition^17^. Neurophysiological predictors of L2 aptitude, especially for learning lexis and communication skills, however, have been reported far less extensively. Among the existing literature, most studies have predicted phonological abilities from anatomical or hemodynamic correlates of L2 proficiency^11,18–20^, but little research has been conducted to characterize a broader disposition for learning languages and even less-so based on pre-training functional organization of the brain, as reflected in resting-state signals. To the best of our knowledge, four studies have been published describing links between the resting state brain and language aptitude or success: in an electroencephalography (EEG) study, Prat et al.^21^ found that in younger adults learning French as a L2, pre-training resting-state (rs) power of oscillations in the beta band (13–29.5 Hz) recorded over right hemisphere electrode sites predicted various global measures of L2 proficiency. Prat et al.^22^ replicated these findings in a study where younger adults learned a programming language, proficiency in which was also predicted by power in beta and low-gamma bands (30–40 Hz). Similarly, Küssner et al.^23^ showed that global pre-training beta power (14–35 Hz) predicted recall scores in younger adults performing a vocabulary-learning paradigm, while Kliesch, Giroud & Meyer^24^ showed that global pre-training power in the lower beta band (13–14.5 Hz) was also predictive of overall L2 development in older adults.

While these studies appear consistent in terms of the predictive power of beta oscillations for L2 development, all defined L2 proficiency mainly in terms of accuracy, when in reality, L2 competence has been shown to consist of at least three subcomponents, namely Complexity, Accuracy and Fluency (CAF)^25^. In order to reliably gauge L2 competence, all three CAF components ought to be tested, as increases in one of them are commonly associated with trade-off effects in others, and performance should be high in all three of them in a proficient speaker of a L2^25^. Understanding neurophysiological substrates of L2 proficiency of older learners in terms of the CAF triad is valuable for two main reasons: (1) It may help us understand individual differences in L2 learning success and develop customized L2 programs for older learners; (2) Understanding the neurocognitive processes that are tapped by L2 learning constitutes a first step towards investigating whether these processes themselves are affected and potentially optimized by the L2 training itself, which would contribute to the discussion of L2-related training interventions to stave-off age-related cerebral and cognitive decline.

As a cognitively challenging form of mental exercise that engages an extensive neural network, L2 learning has been hypothesized to potentially bolster cognitive reserve in old age^26^. The hypothesis is based on studies showing cognitive benefits in older, lifelong bilinguals compared to monolinguals, mainly in terms of working memory and executive functions^27–29^. These findings, however, have recently been called into question on the basis of potential publication biases, unaccounted mediating factors and wider statistical concerns^30,31^, so that the cognitive advantage of lifelong bilingualism is still subject to debate. The few studies that have actually examined longitudinal effects of L2 learning in later adulthood provide little evidence of cognitive improvement^4,32–37^, but no study to date has investigated the changes in functional brain organization driven by L2 learning in older adults that may precede behaviorally significant cognitive changes.

In the present study, we address two questions that may help shed light on the relationship between resting-state activity of the brain and older adults’ disposition to learn a new language. We investigate (1) whether L2 development in older adults can be predicted from electrophysiological pre-training resting-state features and (2) whether the trajectory of L2 proficiency is reflected in changes of resting-state properties over the training. We apply electroencephalography (EEG) measures due to the benefits of its high temporal resolution, which—in conjunction with the spatial resolution—allows differentiating between the frequency bands across which neural assemblies communicate and which have been associated with distinct cognitive functions. We record resting-state EEGs before and after L2 training, since neural, endogenous oscillations as recorded in the awake brain at rest reflect stable aspects of the functional architecture that also underlie evoked oscillatory patterns, and have therefore been identified as an electrophysiological predictor of behavior. We implement measures of functional connectivity, which constitute an ideal correlate of brain function, as they provide information in terms of *how* the brain communicates to achieve a certain behavioral goal, whereas power values—as used in the existing literature—only tell us *that* neurons are firing and in which frequency. Contrary to previous studies, we apply source-localized rather than sensor-level analyses, as this enhances interpretability of the underlying brain sources. Further, our approach is novel in that we investigate the full CAF triad of L2 competence and correlate it with both global and source-localized measures of network connectivity. To characterize global network parameters, we implement Minimum Spanning Trees (MST), which are acyclic subgraphs of a connected brain network that represent the backbone structure of functional connections within the brain. MSTs have been shown to be affected by the aging process and have therefore been proposed as suitable biomarkers for the development of cognitive decline associated with older age^38,39^. By combining MSTs with analyses of localized connections at the source level, we can address whether L2 development correlates with the overall shape of the network, the strength of locally specific connections or both.

The L2 training for this study consisted in a 30-weeks Spanish (L2) training for German-speaking older adults aged 64–74, which comprised 5 weekly hours of L2 practice. CAF were tested at the beginning and at the end of the 30-weeks training period, and a 6-min eyes-closed rs EEG was recorded before the first and after the last session of L2 training. From the recordings, we retrieved connectivity measures between 90 regions of interest, from which we extracted MSTs, and modelled linear regressions to (1) predict individual L2 development, (2) identify cerebral correlates of L2 development, and to do so on both (a) the global (MST) and (b) the source-node level.

## Results

### Behavioral data

**Figure 1:**
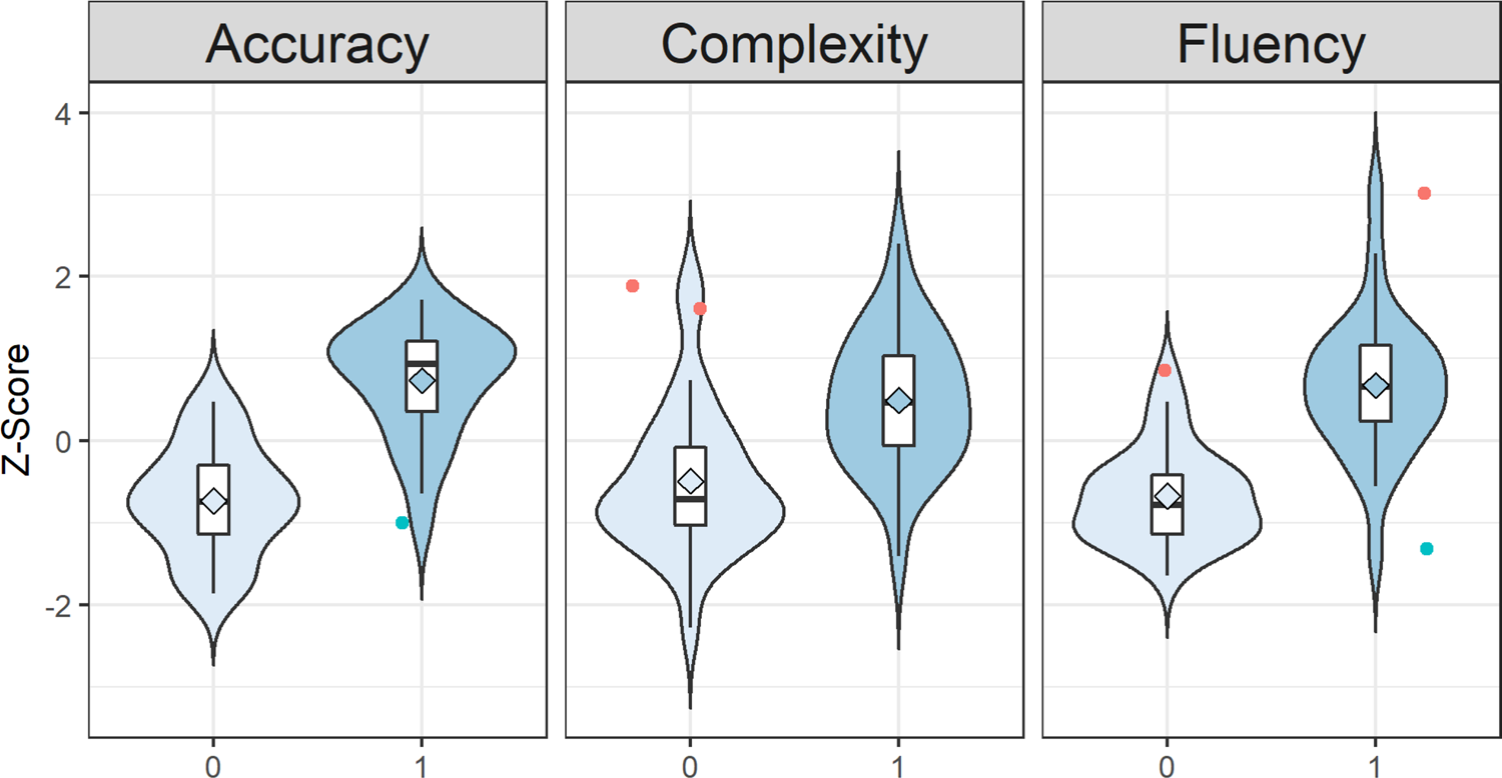
Distribution of z-scores within each measure of L2 proficiency. Violin plots show the probability density of the data, box plots indicate the median and the respective quartiles. The rhombus at the center represents the mean. Blue and red dots are negative and positive outliers, respectively, defined as values above 1.5 IQR from the median.

Separate, one-sided Welch t-tests for dependent samples on standardized L2 scores revealed a significant improvement in all three measures of L2 proficiency. On average, participants’ L2 Complexity improved from *M* = − 0.49, *SE* = 0.92 before the training to *M* = 0.49, *SE* = 0.83, *t*_(54)_ = , *p* < 0.001, *r* = 0.50. Accuracy improved from *M* = − 0.73, *SE* = 0.67 before the training to *M* = 0.73, *SE* = 0.70, *t*_(54)_ = , *p* < 0.001, *r* = 0.74, and Fluency from *M* = − 0.67, *SE* = 0.59 before the training to *M* = 0.67, *SE* = 0.87, t_(47)_ = , *p* < 0.001, *r* = 0.70. Figure 1 shows the distribution of standardized L2 scores for each measure of proficiency before and after the training. Overall and line with Skehan et al.25, there was a relatively strong correlation between L2 Fluency and Accuracy (*r*_*s*_ = 0.59, *p* < 0.001), between Fluency and Complexity (*r*_*s*_ = 0.47, *p* < 0.001) and between Accuracy and Complexity (*r*_*s*_ = 0.68, *p* < 0.001).

### pre-training resting-state EEG predictors of L2 learning

#### Minimum spanning trees

In order to identify global predictors of L2 development, a connectivity matrix of each subject’s pre-training rs EEG activity was extracted and converted into a MST, from which leaf fraction and diameter were extracted (see “Materials and Methods”). An analysis of within-subject consistency of MST parameters revealed medium to large positive correlations between pre and post-training scores for leaf fraction and diameter in the alpha and beta band, which means that MST parameters were reasonably consistent within subjects (see Supplementary Material Figure SF1). In the theta band, however, we observed zero (leaf fraction *r* = 0.09) or even negative correlations (diameter *r* = − 0.32). While we expected a certain amount of change to occur due to the training, we would not expect a learner’s MSTs to be modified to the extent of being unrecognizable between pre and post-training recordings. The lack of MST consistency in the theta band and studies showing theta MSTs to be affected by short-term changes in cognitive states^40^, therefore, led us to remove the entire theta band from further MST analyses. Using a linear regression controlling for baseline levels in L2 development, we found all L2 change scores to be predicted by leaf fraction in the beta band: Complexity *β* = 0.44, *p* = 0.03; Accuracy *β* = 0.22, *p* = 0.04; Fluency *β* = 0.30, *p* = 0.04. The correlations for Complexity and Accuracy remained significant after robustness testing using bootstrapping and remained as a trend for Fluency,but did not survive FDR correction (see Table 1). Thus, independently of the L2 starting level, participants improved their L2 skills more when leaf fraction in the beta band was high, that is, when the respective networks were more star-like than path-like and were characterized by a central organization76. The effects for MST diameter were non-significant (see Supplementary Material Table ST1). The reported connection between rs-EEG and L2 performance was specific to L2 learning, since exploratory analyses did not show any significant relationship between pre-training L2 performance and rs-EEG.

**Table 1:** Results from multiple regressions predicting L2 change based on standardized L2 performance before the training, standardized MST leaf fraction before the training and change in MST leaf fraction over the training.

Frequency Band	L2 Measure		*ΔR2*	*β*	*SE β*	Bootstrap CI (95%)		*p*
						lower	upper	
Alpha	Complexity		.547					
		Intercept		0.976*	**0.166**	**0.176**	**0.970**	**.000**
		L2 pre-training		**− 0.815**	**0.172**	**− 1.261**	**− 0.360**	**.000**
		Leaf fraction pre-train		− 0.148	0.186	− 0.658	0.317	.434
		Leaf fraction change		0.201	0.182	− 0.116	0.632	.282
	Accuracy		.122					
		Intercept		1.471*	**0.093**	**1.142**	**1.682**	**.000**
		L2 pre-training		− 0.119	0.101	− 0.513	0.212	.253
		Leaf fraction pre-train		− 0.027	0.110	− 0.231	0.196	.811
		Leaf fraction change		0.091	0.102	− 0.111	0.303	.380
	Fluency		.118					
		Intercept		1.351*	**0.117**	**1.005**	**1.877**	**.000**
		L2 pre-training		0.088	0.121	− 0.352	0.622	.476
		Leaf fraction pre-train		0.128	0.131	− 0.112	0.527	.338
		Leaf fraction change		0.178	0.127	− 0.026	0.421	.175
Beta	Complexity		.583					
		Intercept		0.944*	**0.162**	**0.286**	**0.879**	**.000**
		L2 pre-training		− 0.792*	**0.162**	− **1.233**	− **0.534**	**.000**
		Leaf fraction pre-train		0.440*	**0.192**	**0.070**	**0.994**	**.032**
		Leaf fraction change		0.241	0.174	− 0.014	0.549	.179
	Accuracy		.308					
		Intercept		1.431*	**0.084**	**1.103**	**1.611**	**.000**
		L2 pre-training		− 0.121	0.088	− 0.472	0.133	.183
		Leaf fraction pre-train		0.222*	**0.101**	**0.017**	**0.496**	**.039**
		Leaf fraction change		0.229*	**0.091**	**0.022**	**0.440**	**.019**
	Fluency		.206					
		Intercept		1.333*	**0.113**	**1.103**	**1.922**	**.000**
		L2 pre-training		0.115	0.117	− 0.230	0.848	.337
		Leaf fraction pre-train		0.295*	0.134	− 0.041	0.687	.039
		Leaf fraction change		0.164	0.123	− 0.170	0.399	.197

Bootstrapped confidence intervals were calculated on the basis of 2000 bootstrap samples. Coefficients marked * are significant (*p* < .05), those marked bold are robust in that they do not contain zero in the bootstrapped confidence intervals. None of the reported correlations between L2 development and MST leaf fraction remained significant when FDR correction for multiple comparisons was applied to the p-values of MST predictors.

#### Connectivity analysis

**Figure 2:**
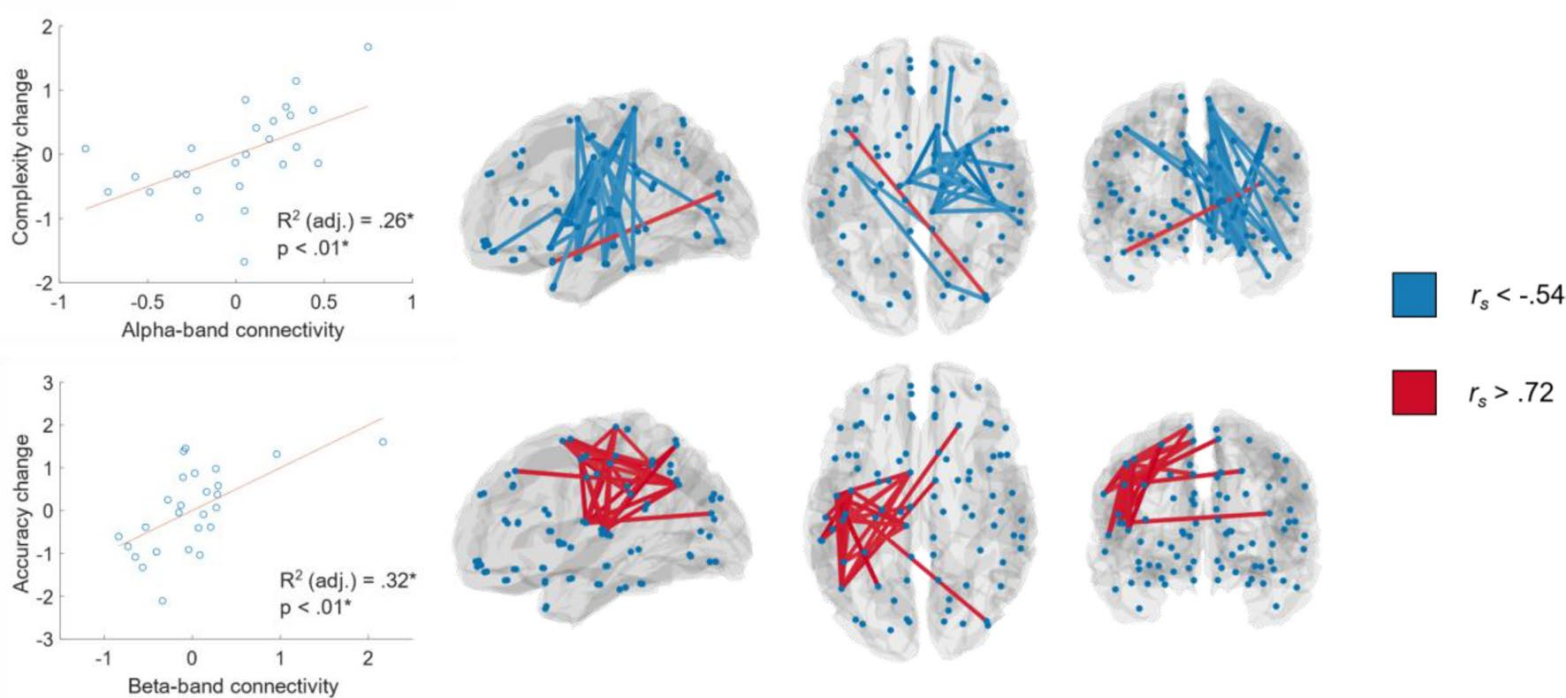
Results of the analysis of the relationship between rs connectivity (pre-learning) and L2 changes (complexity, accuracy, fluency). Top row: rs connectivity in alpha-band and L2 complexity change. Bottom row: rs connectivity in beta-band and L2 accuracy change. Left: Cross-subject correspondence between weighted connectivity scores and L2 change in complexity and accuracy. Right: The 30 strongest edges contributing to above relationships, visualized as correlations coefficients of all edges with respect to the fitted model for the respective relationship. Blue indicates negative weights, implying a decrease in L2 performance with stronger connectivity, while red indicates positive weights and hence increase in L2 performance with increase in connectivity. * Adjusted R-squared and p-values as estimated in the PC-based stepwise regression models (see “Results” and Supplementary Material Tables ST3-4 for details). All relationships depicted also survived permutation testing as a second criterion to establish statistical significance (*p* < .05).

In order to identify localized predictors of L2 development, a stepwise regression was performed on the principal components of the source-localized connectivity matrix and tested for robustness (see “Materials and Methods”). For reasons of consistency with the MST analyses and because overall connectivity in the theta band between pre and post-training only correlated by *r* = 0.198 as compared to *r* = 0.462 in the alpha and *r* = 0.428 in the beta band, findings from the theta band will be omitted. In the alpha band, there was a significant, and mostly negative relationship between RH connectivity and L2 Complexity (*p* < 0.01, *R2* = 0.29), such that learners with low pre-training connectivity between the temporal gyrus, cingulum as well as pre-/post- and paracentral regions showed stronger improvement of L2 Complexity RH (see Fig. 2 and Supplementary Material Tables ST2a and ST2b for model output). In the beta-band, we observed a significant relationship with L2 Accuracy (*p* < 0.01, *R2* = 0.32), albeit in the opposite direction, such that individuals with strong connections within the left pre-/post- and paracentral regions and parts of the temporal and parietal gyri before the training showed stronger increase in L2 Accuracy (see Fig. 2 and Supplementary Material Tables ST3a and ST3b). Similar to the MST analyses, these findings were specific to L2 learning, as no significant relationship was found between connectivity measures and L2 performance before the training.

### Relationship between L2 change and changes in brain connectivity

#### Minimum spanning trees

In order to identify and isolate the relationship between L2 development and changes in RS connectivity, a multiple regression was modelled for each frequency band and L2 measure, predicting L2 change from changes in network characteristics (MST leaf fraction and diameter separately) and controlling for baseline levels in both L2 performance and MST parameters. As shown in Table 1, increases in beta leaf fraction significantly correlated with increases in L2 Accuracy (*β* = 0.23, *p* = 0.02), which remained significant after robustness testing using bootstrapping but did not survive FDR correction. Thus, participants whose RS networks became more star-like and centrally organized by developing increased leaf fraction in beta band showed stronger improvements in L2 Accuracy. Hence, beta leaf fraction was a consistent correlate of Accuracy in that it was both a predictor of L2 development at pre-training and increased in line with L2 Accuracy. The effects for MST diameter were non-significant (see Supplementary Material Table ST1).

#### Connectivity analysis

**Figure 3:**
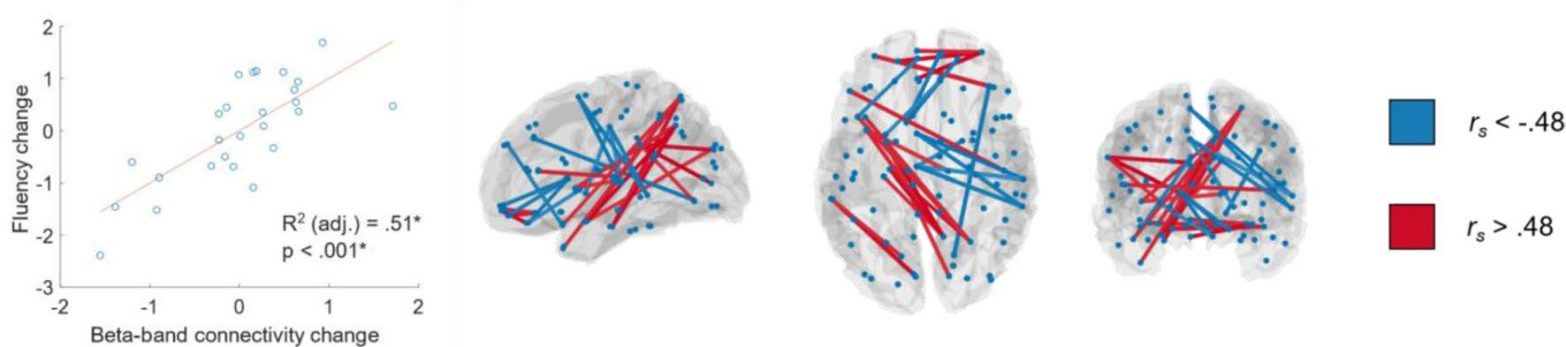
Results of the analysis of the relationship between rs connectivity changes and L2 changes (complexity, accuracy, fluency). Beta-band connectivity changes and L2 changes in fluency were significantly related. Left: Cross-subject correspondence between weighted connectivity scores in beta-band and L2 change in fluency. Right: The 30 strongest edges contributing to this relationship, visualized as in Fig. 2. Blue indicates a decrease in respective L2 performance with stronger connectivity, while red indicates an increase in L2 performance.* Adjusted R-squared and p-values as estimated in the PC-based stepwise regression models (see “Results” and Supplementary Material Tables ST3-4 for details). All relationships depicted also survived permutation testing as a second criterion to establish statistical significance (*p* < .05).

We observed a significant relationship between changes in beta-band connectivity and L2 Fluency (*p* < 0.001, *R2* = 0.57). The respective network comprised both increases and decreases of connectivity. Most connections were cross-hemispheric, the majority of which showed positive relationships (see Fig. 3 and Supplementary Material Tables ST4a and ST4b for more details on outcomes of the model fit and involved edges). Hence, increased LH and cross-hemispheric connectivity in the beta band was both a predictor and correlate of L2 development.

## Discussion

The aim of the present study was to identify predictors and correlates of L2 learning in older adults based on individual differences in global and localized patterns of functional connectivity. This is the first study to investigate electrophysiological correlates of the CAF framework and to provide evidence of global and of localized network characteristics in the rs as they relate to changes in CAF. We found rs-EEG connectivity to be both a predictor of and affected by changes in L2 proficiency. In both cases, significant effects were identified on both the global network level (MSTs) as well as on the level of local connections, and results were found to be consistent across participants, as confirmed via robustness tests. Both the alpha and the beta band showed locally-specific patterns of connectivity that predicted L2 development, but only in the beta band, global network parameters (i.e. leaf fraction) were significant predictors, too. It was also in the beta band where we observed modifications of connectivity that significantly correlated with increases in L2 development, such that changes in L2 fluency were reflected in local connectivity changes and changes in L2 accuracy were reflected in global beta network changes.

EEG predictors of L2 acquisition in the brain at rest are of particular interest for a number of reasons. For one, they help us understand older learners’ individual differences in their ability to learn a new language even before training starts. This in turn, makes functional rs connectivity an important foundation for individualizing L2 instruction for older learners. At the same time, these rs correlates allude to an underlying neurofunctional mechanism that applies to varying measures of L2 proficiency. Here, we gauged L2 proficiency using measures of complexity, accuracy and fluency (CAF), which are known to follow varying or even contradictory trajectories in L2 learners^25^. Despite this, all three of the L2 measures assessed were predicted by localized and by global (MST) patterns of connectivity in the alpha and beta band, which confirms that the entire CAF triad is encoded in the resting brain. As our results have confirmed, however, rs-EEG connectivity does not only mark a predisposition for L2 learning, but is itself affected by the increase in L2 proficiency, which constitutes a first step towards understanding long-term effects of L2 training on rs connectivity. Since the rs is a reliable predictor of cognitive wellbeing and healthy aging^41–43^, L2 training-induced transfer effects may provide first evidence of beneficiary neuroplasticity effects that may eventually lead to domain-general cognitive stabilization or help prevent or stave off age-related cognitive declines.

### Beta connectivity and L2 learning

Our finding of beta connectivity being both a predictor and a correlate of L2 development echoes previous studies that found a consistent relationship between beta power or coherence and L2 development^21,23,24^. Beta oscillations have repeatedly been linked to attentional performance and endogenously-driven top-down attention^44–46^, and it is precisely attentional resources that the CAF triad has been argued to compete for^25^. In addition, the allocation of attentional resources has repeatedly been identified as a key element to acquiring a L2^16,47^. Therefore, the involvement of the beta band may point to domain-general attentional resources that facilitate the noticing and uptake of new L2 information before it can be processed and stored in working memory and long-term memory. While the general role of beta oscillations in L2 learning has been reported several times, our study is the first to distinguish between global and localized connectivity correlates of L2 development. By doing so, we can differentiate whether L2 learning is mainly facilitated by the strength of connectivity between particular nodes in the cerebral network or whether it is determined by the overall shape of the network that may allow information to travel more or less efficiently. For the beta band, we identified predictors and correlates of L2 learning on both the global and the localized level.

On the level of MSTs, higher leaf fraction predicted better L2 attainment of all three measures of L2 proficiency and increased in line with improvements of L2 Accuracy. Leaf fraction is a measure of a network’s central organization, such that a higher leaf fraction characterizes a network whose communication is largely dependent on hub nodes^81^. This means that beta networks with a more central organization were beneficial for L2 learning, and the beta networks became more centrally organized as L2 Accuracy increased. In line with the overall involvement of beta in attentional processes, previous studies have found long-range rs connections in the beta band—particularly between fronto-parietal regions—to be detrimental for cognitive performance. Fleck et al.^48^, for instance, found that working memory performance was decreased in older adults who showed increased coherence between fronto-parietal electrodes in the beta band. Similarly, Rogala et al.^49^ could show that in younger adults, increased connectivity between fronto-parietal electrodes during a visual attention task and at rest were associated with decreased attentional performance, which the authors interpreted as a sign of decreased capacity for network configuration and of impaired cost-effective strategies^49^. Hence, a beta network with a high leaf fraction could be indicative of cost-effective allocation of attentional resources in the face of the shifting requirements during L2 training.

Further, our study is the first to identify a relationship between L2 performance and source localized functional beta networks, allowing us to map them onto cognitive processes and brain networks characterized in previous studies. For instance, the frontal network that, here, showed an increase in connectivity and the weakened network in medial and fronto-temporal long-range beta connections as a function of increased L2 proficiency intersects with the beta networks predicting working memory performance (e.g. Digit Span Forward, Digit Span Sequencing) in older adults^48^. Even more evident, however, is the beta network predicting development of L2 accuracy, which included LH parietal, central and paracentral as well as sensorimotor areas. These areas characterize the typical beta rs network, and while they are commonly involved in motor skills, they have also been associated with performance in working memory, inhibition, attention and, importantly, speech tasks^50^. The fact that this left-lateralized temporo-parietal network was associated with L2 Accuracy, in particular, is in line with studies showing similar activations during morpho-syntactic and morpho-semantic processing, both of which were strongly involved in our measure of L2 Accuracy^51,52^. Apart from cognitive and linguistic skills themselves, LH parietal beta power, in particular, has been associated with cognitive styles, too, in that it has been found to be more pronounced in people using insightful problem-solving rather than analytic problem-solving^53^. This finding is consistent with studies showing that older L2 learners benefit more from implicit than explicit instructional feedback^54^, the latter of which would typically be integrated using analytic problem-solving and therefore potentially disfavored by older learners. Finally, at the subsegmental auditory level, LH parietal and temporal beta at rest has been linked to better temporal discrimination^55^ and speech-in-noise recognition due to a higher efficacy of phoneme processing^56^, both of which are in line with studies identifying phonetic discrimination as a key predictor of L2 development^57^.

### Alpha connectivity and L2 learning

While the alpha rhythm is the brain’s most dominant rhythm and has been shown to decrease in power with age^43^, increased alpha connectivity is not always beneficial^50^ and has been associated, for instance, with excessive self-focus and rumination^58^. Here, we identified a RH alpha network comprising temporal, medial and parietal regions whose connections were particularly weak in individuals who showed a higher degree of L2 development. In line with the beta network that has been attributed to cognitive top-down processes, hyperconnectivity in the alpha band between RH fronto-temporal areas, as observed in the present study, has also been associated with decreased cognitive function (e.g. immediate recall, semantic fluency)^59^. In the case of the alpha band, however, there appears to be an additional dimension of affective and motivational aspects that have commonly been associated with networks of hyperconnectivity. For example, increased relative RH frontal alpha activity has been associated with increased anxiety, decreased impulsivity^60^ and dysphoria^61^. Unsurprisingly, linguistic complexity has been shown to be reduced as a function of anxiety and introversion^62^, which would explain its association with alpha connectivity in our study, whereas Accuracy was only predicted by the beta network reflecting cognitive control. Finally, L2 Complexity is also a measure of linguistic creativity, as it measures the amount of variation in the learners’ utterances, and creativity, in turn, has been found to activate similar networks as the one reported here, including right-hemispheric temporo-parietal, medial frontal and cingulate cortices^63,64^.

## Summary

The beta networks identified in the present study largely overlap with domain-general networks of cognitive control, cognitive strategies and subsegmental processing, while the alpha networks more likely reflect socio-affective and motivational preconditions. Interestingly, despite their often temporally divergent course, all three measures of the CAF triad were predicted by rs features in the beta band, but only L2 Complexity showed a relationship with pre-training alpha connectivity, too. Given that both L2 Complexity and rs connectivity in the alpha band have been associated with anxiety, rumination and other motivational factors, it is likely that proficiency in this L2 measure resulted as an interaction of cognitive and socio-affective aspects, whereas the Accuracy and Fluency relied more heavily on top-down cognitive resources. Interestingly, our measures of rs connectivity were not only predictors of the learning outcome but were themselves affected by changes in L2 proficiency. This confirms that plasticity is a characteristic of the aging brain, too, and highlights potential implications of L2 learning for cognitive reserve and the prevention of age-related cognitive declines^65^. As our results have shown, both global network analyses (MSTs) and local patterns of connectivity can be informative in this regard, as they were both complementary and confirmatory in the present study. For the beta band, both global and localized connectivity predicted changes in L2 Complexity, but development of L2 Fluency was only predicted by global beta network characteristics.

## Limitations

Since this study was conducted in Switzerland, the local language system should be borne in mind when interpreting the present findings. Due to there being four official languages in Switzerland, a certain knowledge of Romance languages (i.e. French, Italian) was inevitable, which explains why several learners showed non-zero baseline L2 scores even in the absence of prior Spanish training. In addition, given the test modalities used to assess L2 proficiency, non-zero baseline scores can also be produced with very low L2 knowledge: In the case of L2 Accuracy, non-zero scores likely resulted from the fact that some of the tasks were receptive, so that previous contact with other languages and a certain chance level may have been beneficial at study onset. Similarly, Fluency was already non-zero once a few words had been uttered, and all participants produced at least a handful of Spanish words in each of the recordings. Accordingly, Complexity is simply a measure of the variety of lexemes used, so that by producing two different words, participants already obtained a Complexity score above zero. However, since baseline performance was included in all our models, we controlled for the influence the starting level can have on the ensuing learning slope, so that the brain-behavior relationship identified in the present study can be interpreted independently of pre-existing L2 knowledge.

## Conclusion

This study constitutes a first important step towards understanding individual differences in later learners’ ability to acquire a new language based on electrophysiological underpinnings and their involvement in domain-general cognitive and affective processes. These results confirm that the capacity to acquire a L2 is encoded in the resting brain and that rs activity can be an informative, task-free way of recording behaviorally meaningful individual traits. Future studies could further investigate how exactly the relationship between rs connectivity and L2 development is mediated by cognitive ability or socio-affect and how the predictive power of these rs parameters changes with increasing L2 proficiency. Furthermore, it would be interesting to investigate where the here-observed rs changes are impactful enough to generate improvements in cognitive performance given a certain training intensity and duration, particularly in individuals who already manifest pathologically decreased cognitive performance, as is the case in individuals suffering from dementia. Finally, the present study is based on a purely correlational design, and future studies should address the effect of L2 training on functional rs connectivity when compared to other forms of training.

## Materials and methods

### Participants

We recruited 31 quasi-monolingual participants aged 64 to 74 (see Table 2), who were native speakers of (Swiss) German (see Kliesch & Pfenninger^3^) with no previous knowledge of Spanish and no more than school knowledge of any other language. Participants were healthy, i.e. they did not report any psychological or neurological disorders or learning disabilities. In order to ensure comparability of cognitive capacities and exclude individuals suffering from cognitive impairment, a Montreal Cognitive Assessment (MoCA^66^ was administered in a screening session, and individuals with scores lower than 26/30 were excluded. We further excluded professional musicians (i.e. playing an instrument for more than 6 h per week), as musical expertise has been shown to facilitate L2 learning^67^. Participation in the study was voluntary and all participants gave their informed consent at the beginning of the screening. To ensure that participants showed similar levels of motivation during the study, we assessed their motivation and overall wellbeing through weekly-administered questionnaires using slider scales from 1–100 (higher = better, see Kliesch & Pfenninger^3^). Out of the 31 individuals who joined the study, three withdrew their participation over the course of the training, and two further had to be excluded due to file damage of the EEG recordings. The study was approved by the ethics committee of the Philosophical Faculty of the University of Zurich (Project ID: 18.6.3) and all experiments were performed in accordance with relevant guidelines and regulations.

**Table 2:** Participant Information (N = 26).

Statistic	Age	Education (n years)	Av. Training Motivation	Av. Wellbeing
Median	67	12	69.23	73.54
Min	64	8	46.86	47.62
Max	74	18	97.41	98.19

Possible scores for Training Motivation and Wellbeing ranged between 1–100 and were indicated on a slider scale. Both Training Motivation and Wellbeing showed ceiling effects, indicating that participants were highly motivated during the training.

### L2 proficiency

L2 proficiency was assessed weekly by three written tests and a 5-min semi-guided oral interview in the L2., from which three overarching measures of L2 performance were derived: Complexity, Accuracy and Fluency (CAF)^25^. The CAF framework is based on a Trade-off Hypothesis assuming limited attentional resources and working memory, which predicts that committing attention to one of the three areas may cause lower performance in others, and that L2 proficiency is characterized by high performance in all three areas. For details on each of the tests, see Kliesch & Pfenninger^3^.

#### Accuracy

Accuracy, i.e. error-free L2 performance, was calculated from a C-Test measuring integrative L2 skills^68^, a grammatical comprehension test^69^, a lexical comprehension task, morphosyntactic accuracy in the oral interviews and target-like accuracy (i.e. the avoidance of non-Spanish words) in the oral interviews. Each accuracy score was z-normalized prior to calculating the mean over all five measures.

#### Complexity

Complexity is the use of challenging and diverse language^25^ and was assessed via the Measure of Textual Lexical Diversity^70^, which measures the ratio of the number of different lexemes to the overall number of words/text units.

#### Fluency

Fluency was defined in terms of speech rate and was measured as the number of words/text units produced within the 5-min interview independent of whether they were correct or not.

### Experimental procedure/training

A resting-state EEG was recorded prior to the L2 training and again no later than one week following the training. The L2 training took place over 30 weeks, during which participants learned Spanish via both in-class communicative language teaching and autonomous practice at home using the language learning software Duolingo. Duolingo is a gamified and mobile language tool whose effectiveness in improving L2 proficiency has been documented in several studies^71^.Participants were asked to complete 450XP (experience points) per week in Duolingo, for which participants needed approximately 2–3 h. The weekly sessions were taught by a Spanish instructor and took place over 90 min. They were conducted in groups of 5–8 subjects and focused on oral practice, communication and grammar. The in-class sessions further included time slots for the L2 tests and oral interviews, which were carried out each week and by research assistants other than the L2 instructor (see Kliesch & Pfenninger^3^ for details). The group affiliation of each learner remained the same throughout the training, as did the instructor. An exploratory analysis using the intraclass correlation coefficient (ICC1) did not show evidence of different L2 development across classrooms, so that classrooms were omitted from all further analyses.

### EEG data acquisition and pre-processing

Six minutes of eyes-closed resting-state EEG were obtained using 64 active channel actiCap electrode caps coupled to a BrainAmp DC amplifier system (Brain Products Gmbh, Gilching, Germany) at a sampling rate of 1000 Hz. The Ag/AgCl electrodes were places according to the 10/5 position system^72^, using the frontal electrode FCz as online reference and AFz as ground electrode. All impedances were kept below 15 kΩ. For all pre-processing steps, we used the FieldTrip toolbox in MATLAB^73^. Data were filtered off-line with a low-pass filter of 80 Hz, a high-pass filter of 0.5 Hz and a band stop filter between 49.5 and 50.5 Hz. Noisy channels were removed following visual screening. For the ICA, data were re-referenced to the average reference and high-pass filtered at 1 Hz. Artefactual components (blinks, saccades, EKG artefacts) were removed after visual inspection. Finally, noisy channels were interpolated using spline interpolation, and the continuous EEG was then segmented into trials of 3 s, removing trials containing gross artefacts manually.

### Functional connectivity analysis

While most studies bin frequency into fixed bands, researchers have advocated applying individualized frequency bands given the known variability of the individual alpha frequency (IAF) with advancing age^74^ and the reduced connectivity when using conventional spectral boundaries in older adults^75^. Hence, to obtain functional connectivity matrices of each participant, we first estimated the IAF for each subject by applying the automated peak detection developed by Corcoran et al.^76^. Participants who did not show a clear peak were assigned the median IAF of 9.69. Frequency bands were defined with respect to each participant’s IAF^74^, with IAF representing 0 on a relative frequency spectrum. Theta was defined from − 6 to − 3 (i.e. 6 to 3 Hz below the IAF), alpha from − 2.5 to + 2.5 and beta from + 3 to + 19. A time–frequency analysis was performed within each of the band and the signal’s sources were estimated via a partial canonical coherence beamformer approach^77^. To reduce biases towards the center of the head, we computed the neural activity index (NAI) by dividing the estimated power at each grid point by an estimate of the noise based on the smallest eigenvalue of the cross-spectral density matrix^78^. Subsequently, functional connectivity between each of the grid points was calculated as the imaginary part of coherency^79^, which has been shown to be insensitive to false connectivity arising from volume conduction and therefore produces more objective but also more conservative estimates than other measures of connectivity. Finally, the source-resolved connectivity matrices were anatomically parcellated according to the AAL atlas^80^, which resulted in 90 anatomical volumes of interest (45 in each hemisphere). Connectivity matrices between each of the 90 anatomical volumes were used for further analyses.

### Minimum spanning trees

**Figure 4:**
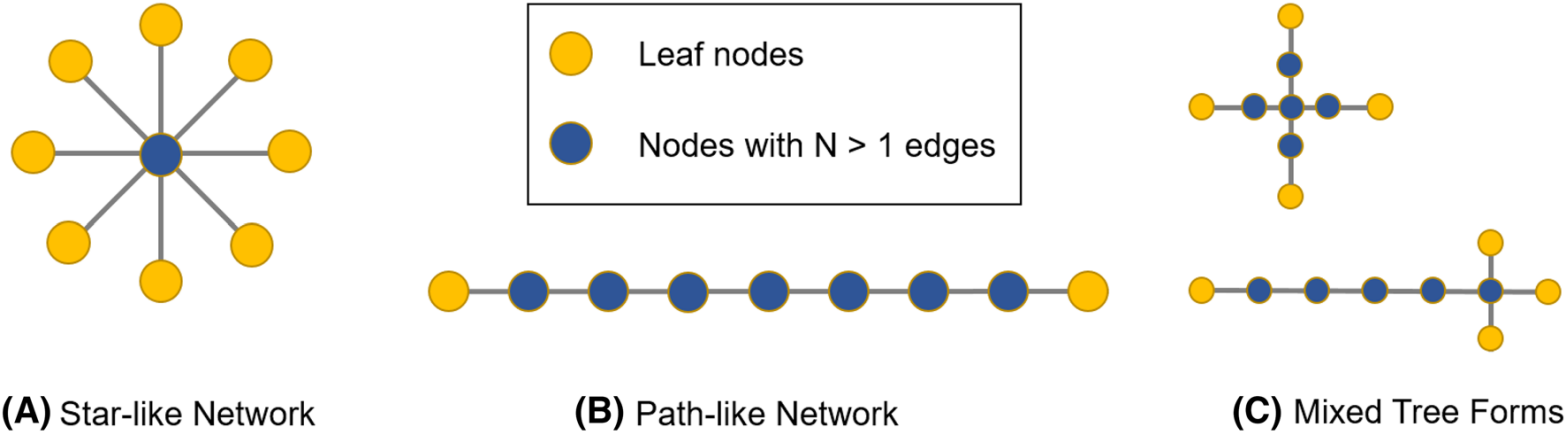
Schematic illustration of Minimum Spanning Trees (along the lines of Stam et al.^83^). Different configurations consisting of N = 9 nodes and N = 8 edges. (**A**) Tree with shortest possible diameter and maximum number of leaves. (**B**) Tree with longest possible diameter and lowest number of leaves. (**C**) Mixed tree forms with a diameter of 4 (top) and 6 (bottom) and a leaf number of 4 in both cases. For our analyses, diameter and leaf fraction were standardized by the total number of edges and maximum possible leaf fraction, respectively.

In order to identify the characteristics of each subject’s connectivity matrix as a brain graph, we extracted Minimum Spanning Trees (MST), which is a subnetwork containing the strongest connections from the set of all available weighted connections without ever forming loops^81^. MSTs are preferable to traditional graph theory analyses, as they are unaffected by the thresholding problem, which can cause differing sample sizes between subjects and even lead to contradictory findings^82^. A MST was obtained for each participant and within each frequency by ranking all connection weights from the original connectivity matrices from highest weight to lowest weight. The two strongest connections were used to start each MST and the next strongest connections were added as long as they did not form loops with previous edges. This approach will always result in exactly one edge less than number of nodes. Finally, for each MST, the two measures leaf fraction and diameter were extracted (see Fig. 4). The leaf fraction characterizes the number of nodes that only have one connection in the tree and is normalized by the maximum number of possible leaves, ranging from 0 (path-like network) to 1 (star-like network). The diameter is the largest distance between any two nodes, normalized by the total number of connections in the tree, and ranges between 0 (star-like network) and 1 (path-like network). While the two parameters cannot be translated directly into conventional measures of graph theory (e.g. clustering coefficient and path length), they have been shown to be at least as sensitive, or more sensitive than, conventional graph theory approaches in the characterization of network changes^83^.

### Statistical analyses

Each of the three L2 measures was standardized prior to entering the models such that a z-score of zero represents an average L2 performance across the two time points and higher z-scores signify better L2 proficiency. The analyses of data on L2 proficiency were performed by using a one-tailed t-test for dependent samples. The relationship between L2 proficiency change scores with (1) MST parameters before the training and (2) MST parameter change scores was assessed via linear regression, in which baseline levels of L2 proficiency and MST parameters were inserted as z-normalized predictors so as to account for the regression to the mean^84^. According to this phenomenon, change scores tend to be negatively related to baseline values and since neither the L2 scores nor the values for network topology were zero at study onset, that had to be included in the same model as the change scores. Separate models were created for MST leaf fraction and diameter. Exploratory analyses revealed no significant correlation between background variables (education, wellbeing, training motivation, gender) and either behavioral outcomes (Supplementary Material Figure SF2) or resting-state connectivity (Supplementary Material Figure SF3). To avoid overfitting and to account for non-normality in the background variables, they were not included the further analyses. For all models, we set the alpha level at *ps* < 0.05 and controlled for multiple comparisons by applying False Discovery Rate (FDR) correction. We used R (3.6.1) for behavioral and MST analyses and used MATLAB (2017b) for Principal Component Analysis and Stepwise Regressions on the raw connectivity matrices.

The relationships between connectivity and L2 proficiency were evaluated as follows. Per frequency band and after concatenating all edges per subject, PCA was carried out on the full connectivity data, i.e. on a 26 × 8010 (subject x connectivity) matrix, and the first six components, explaining 62.3% of the variance in the pre-training connectivity, were retained for further analysis. In order to explain a similar amount of variance in the difference scores (post minus pre-training), the number of retained components had to be increased to 10, in order to explain 65% of the variance in the alpha and beta bands. Those models that were significant (*ps* < 0.05 compared to constant model) in this first step, i.e. the stepwise regression, were subsequently subjected to a permutation statistical approach where we swapped subject labels and then subjected those permutations to stepwise regression with identical parameters as before, running n = 1000 permutations per relationship. Resulting null models were then used to compare the outcome of the significant stepwise regression models (i.e. their p-value of the overall F-statistics) to the permutation-based p-values. Only models that were both significant in the stepwise regression and which were also doing better than the top 5% of the permutation based stepwise regressions were considered significant. This two-step validation approach addresses concerns of the potential lack of robustness of approaches using variable elimination such as stepwise regression [Smith, 2018]. Separate models were computed for respective frequency bands, i.e. for alpha and beta frequency range, and for each of the L2 measures. To visualize the association strength of the full connectivity edges to the above-identified modes, we correlated the connectivity score of each edge (its variation across subjects) with the established mode from the models, that is, the subject-wise PCs weighted by the beta coefficients resulting from the regressors of the significant models.

## Supplementary Information

Supplementary Information.

## Data Availability

The datasets generated during and/or analyzed during the current study are available from the corresponding author on reasonable request.
