# Supplementary material for: Global and localized network characteristics of the resting brain predict and adapt to foreign language learning in older adults
Source: Sci Rep. 2022 Mar 7;12:3633. doi: 10.1038/s41598-022-07629-y (PMC8901791; doi:10.1038/s41598-022-07629-y)
Supplement: Supplementary file 1 — Supplementary Information. [file 41598_2022_7629_MOESM1_ESM.docx]

**Figure SF1**: Correlation matrix of MST parameters between pre and post-training sessions for the theta, alpha and beta bands.


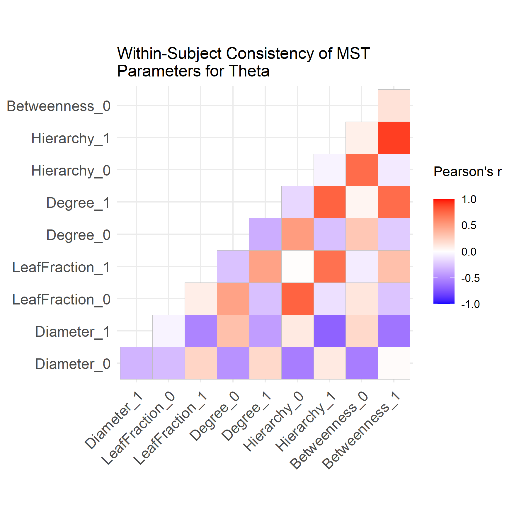

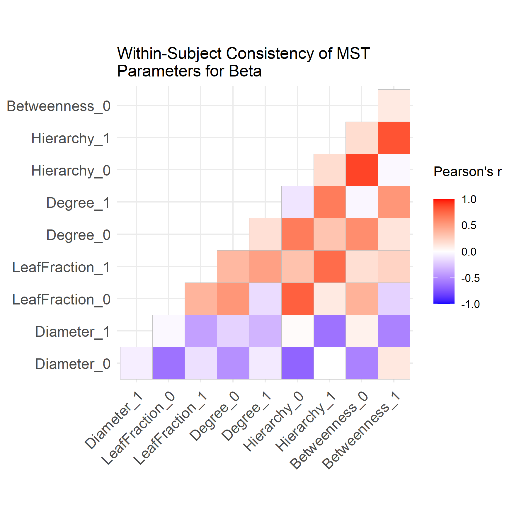

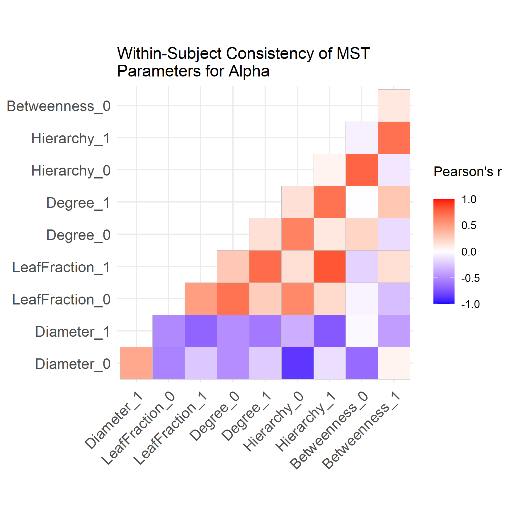


**Figure SF2:** Correlation matrix of L2 development and background variables.


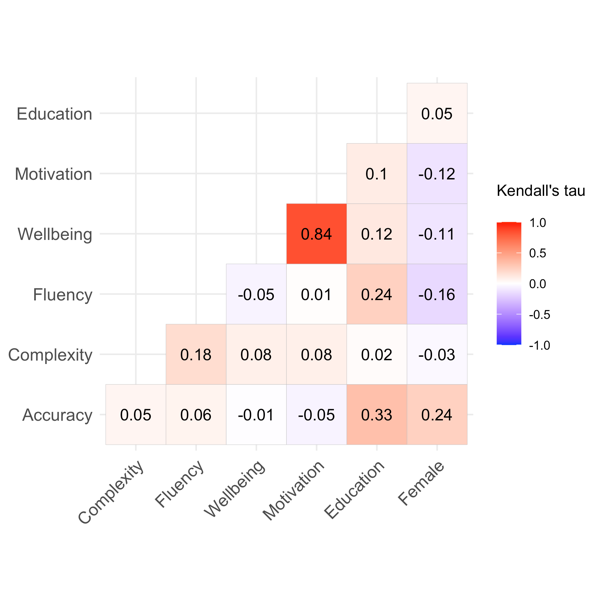


*Note: Education = education years; Motivation = mean training motivation across the entire training period; Wellbeing = mean wellbeing across the entire period; Fluency, Complexity, Accuracy = change scores post-training minus pre-training; Female = gender (female = 1; male = 0). None of the reported correlations between L2 development and background variables were significant.*

**Figure SF3:** Correlation matrix of changes in brain network characteristics and background variables.


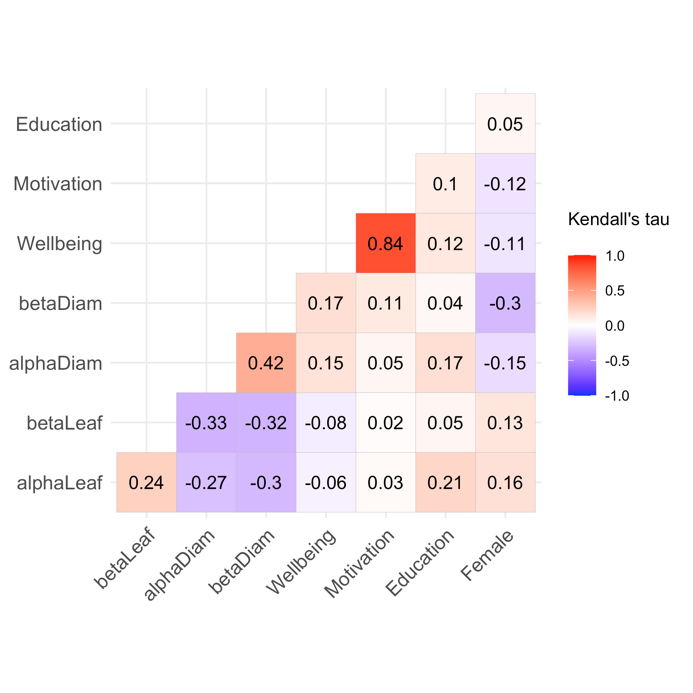


*Note: Education = education years; Motivation = mean training motivation across the entire training period; Wellbeing = mean wellbeing across the entire period; betaDiam = diameter beta band; alphaDiam = diameter alpha band; betaLeaf = leaf fraction beta band; alphaLeaf = leaf fraction alpha band; all resting-state parameters represent post-training minus pre-training scores. None of the reported correlations between resting-state development and background variables were significant.*

**Table ST1**

| *Results from Multiple Regressions Predicting L2 Change Based on Standardized L2 Performance Before the Training, Standardized MST Diameter Before the Training and Change in MST Diameter Over the Training.* | | | | | | | | | |
| --- | --- | --- | --- | --- | --- | --- | --- | --- | --- |
| Frequency Band | L2 Measure |  | *ΔR2* | *β* | *SE β* | Bootstrap CI (95%) | | *p* | |
|  |  |  |  |  |  | lower | upper |  |  |
| Alpha | Complexity |  | .511 |  |  |  |  |  | |
|  |  | Intercept |  | 1.038 | 0.180 | 0.270 | 0.934 | .000 | |
|  |  | L2 pre-training |  | -0.793 | 0.178 | -1.273 | -0.617 | .000 | |
|  |  | Diameter pre-train. |  | -0.053 | 0.209 | -0.586 | 0.473 | .802 | |
|  |  | Diameter change |  | 0.155 | 0.201 | -0.304 | 0.508 | .447 | |
|  | Accuracy |  | .088 |  |  |  |  |  | |
|  |  | Intercept |  | 1.478 | 0.099 | 1.130 | 1.685 | .000 | |
|  |  | L2 pre-training |  | -0.149 | 0.104 | -0.531 | 0.174 | .167 | |
|  |  | Diameter pre-train. |  | -0.069 | 0.118 | -0.254 | 0.209 | .567 | |
|  |  | Diameter change |  | -0.01 | 0.109 | -0.191 | 0.259 | .929 | |
|  | Fluency |  | .144 |  |  |  |  |  | |
|  |  | Intercept |  | 1.361 | 0.120 | 0.985 | 1.880 | .000 | |
|  |  | L2 pre-training |  | 0.067 | 0.119 | -0.436 | 0.625 | .577 | |
|  |  | Diameter pre-train. |  | -0.215 | 0.140 | -0.544 | 0.075 | .140 | |
|  |  | Diameter change |  | -0.023 | 0.132 | -0.281 | 0.251 | .863 | |
| Beta | Complexity |  | .525 |  |  |  |  |  | |
|  |  | Intercept |  | 0.906 | 0.187 | 0.268 | 0.905 | .000 | |
|  |  | L2 pre-training |  | -0.727 | 0.183 | -1.224 | -0.527 | .001 | |
|  |  | Diameter pre-train. |  | -0.367 | 0.264 | -0.882 | 0.303 | .177 | |
|  |  | Diameter change |  | -0.202 | 0.182 | -0.499 | 0.243 | .277 | |
|  | Accuracy |  | .083 |  |  |  |  |  | |
|  |  | Intercept |  | 1.503 | 0.105 | 1.106 | 1.681 | .000 | |
|  |  | L2 pre-training |  | -0.143 | 0.103 | -0.506 | 0.189 | .177 | |
|  |  | Diameter pre-train. |  | 0.065 | 0.143 | -0.287 | 0.348 | .651 | |
|  |  | Diameter change |  | 0.052 | 0.103 | -0.145 | 0.292 | .619 | |
|  | Fluency |  | .051 |  |  |  |  |  | |
|  |  | Intercept |  | 1.388 | 0.133 | 0.990 | 1.882 | .000 | |
|  |  | L2 pre-training |  | 0.108 | 0.124 | -0.372 | 0.740 | .394 | |
|  |  | Diameter pre-train. |  | 0.121 | 0.178 | -0.216 | 0.513 | .504 | |
|  |  | Diameter change |  | 0.045 | 0.124 | -0.172 | 0.425 | .719 | |
| *Note*: Bootstrapped confidence intervals were calculated on the basis of 2000 bootstrap samples. Coefficients marked with an asterisk are robust in that they do not contain zero in the bootstrapped confidence intervals. None of the reported correlations between L2 development and MST leaf fraction remained significant when FDR correction for multiple comparisons was applied to the p-values of MST predictors. | | | | | | | | | |
|  | | | | | | | | |  |

**Table ST2a**

Stepwise regression for pre-training alpha connectivity predicting Complexity changes.

|  | *β* | *SE β* | *t*(24) | *p*(β) | *F* | *p*(F) | *R^2^* (adj.) |
| --- | --- | --- | --- | --- | --- | --- | --- |
|  |  |  |  |  | 9.7 | < 0.01 | 0.26 |
| Intercept | 0.00 | 0.12 | 0.00 | 1.00 |  |  |  |
| PC5 | -0.39 | 0.12 | -3.11 | < 0.01 |  |  |  |

**Table ST2b**

Top 30 edges contributing to the mode predicting Complexity changes based on pre-training alpha connectivity.

| Edges involved in mode | | Association strength |
| --- | --- | --- |
| Pallidum_R | Postcentral_R | -0.70 |
| Cingulum_Mid_L | Olfactory_R | -0.69 |
| Cingulum_Mid_R | Olfactory_R | -0.66 |
| Putamen_R | Postcentral_R | -0.66 |
| Putamen_R | Paracentral_Lobule_R | -0.64 |
| Amygdala_R | Cingulum_Mid_L | -0.62 |
| Pallidum_R | Paracentral_Lobule_R | -0.62 |
| Thalamus_R | Amygdala_R | -0.62 |
| Thalamus_R | Paracentral_Lobule_R | -0.61 |
| Caudate_R | Frontal_Sup_Orb_R | -0.60 |
| Amygdala_R | Cingulum_Mid_R | -0.60 |
| Thalamus_R | ParaHippocampal_R | -0.60 |
| Paracentral_Lobule_R | Hippocampus_R | -0.60 |
| Pallidum_R | Caudate_R | -0.60 |
| Temporal_Inf_R | Cingulum_Mid_L | -0.59 |
| Amygdala_R | Supp_Motor_Area_R | -0.58 |
| Calcarine_R | Precentral_L | -0.58 |
| Supp_Motor_Area_R | Rolandic_Oper_R | -0.58 |
| Thalamus_R | Olfactory_R | -0.58 |
| Temporal_Inf_R | Paracentral_Lobule_R | -0.58 |
| Paracentral_Lobule_R | ParaHippocampal_R | -0.58 |
| Temporal_Mid_R | Supp_Motor_Area_R | -0.56 |
| Occipital_Inf_R | Calcarine_R | -0.55 |
| ParaHippocampal_R | Cingulum_Mid_R | -0.55 |
| Thalamus_L | Amygdala_R | -0.55 |
| Temporal_Pole_Mid_R | Cingulum_Mid_L | -0.55 |
| Hippocampus_R | Cingulum_Mid_R | -0.54 |
| Cingulum_Post_R | Precentral_L | -0.54 |
| Temporal_Inf_R | Cingulum_Mid_R | -0.54 |
| Temporal_Pole_Sup_L | Occipital_Mid_R | 0.58 |

**Table ST3a**

Stepwise regression for pre-training beta connectivity predicting Accuracy changes.

|  | *β* | *SE β* | *t*(24) | *p*(β) | *F* | *p*(F) | *R^2^* (adj.) |
| --- | --- | --- | --- | --- | --- | --- | --- |
|  |  |  |  |  | 6.88 | < 0.01 | 0.32 |
| Intercept | 0.00 | 0.16 | 0.00 | 1.00 |  |  |  |
| PC3 | 0.46 | 0.16 | 2.89 | 0.01 |  |  |  |
| PC5 | 0.37 | 0.13 | 2.32 | 0.03 |  |  |  |

**Table ST3b**

Top 30 edges contributing to the mode predicting Accuracy changes based on pre-training beta connectivity.

| Edges involved in mode | | Association Strength |
| --- | --- | --- |
| Angular_L | Precentral_L | 0.72 |
| Postcentral_L | Supp_Motor_Area_L | 0.73 |
| Paracentral_Lobule_L | Parietal_Inf_L | 0.73 |
| SupraMarginal_L | Supp_Motor_Area_L | 0.73 |
| Postcentral_L | Cingulum_Mid_L | 0.73 |
| Paracentral_Lobule_L | Postcentral_L | 0.74 |
| Parietal_Inf_L | Supp_Motor_Area_L | 0.74 |
| Angular_L | Supp_Motor_Area_L | 0.74 |
| Occipital_Mid_R | Rolandic_Oper_L | 0.75 |
| Heschl_L | Parietal_Inf_L | 0.75 |
| Angular_L | Frontal_Sup_R | 0.76 |
| Supp_Motor_Area_L | Precentral_L | 0.76 |
| Paracentral_Lobule_L | Precentral_L | 0.76 |
| Temporal_Sup_L | Postcentral_L | 0.76 |
| Paracentral_Lobule_L | Rolandic_Oper_L | 0.77 |
| SupraMarginal_L | Postcentral_L | 0.77 |
| Temporal_Sup_L | Precentral_L | 0.77 |
| Heschl_L | Angular_L | 0.78 |
| Angular_L | Supp_Motor_Area_R | 0.78 |
| Angular_L | Rolandic_Oper_L | 0.78 |
| Postcentral_L | Precentral_L | 0.78 |
| Heschl_L | Postcentral_L | 0.78 |
| Heschl_L | Precentral_L | 0.80 |
| Parietal_Sup_L | Rolandic_Oper_L | 0.80 |
| Parietal_Inf_L | Rolandic_Oper_L | 0.81 |
| Rolandic_Oper_L | Precentral_L | 0.81 |
| Parietal_Inf_L | Precentral_L | 0.81 |
| Postcentral_L | Rolandic_Oper_L | 0.82 |
| Heschl_L | Parietal_Sup_L | 0.83 |
| SupraMarginal_L | Precentral_L | 0.84 |

**Table ST4a**

Stepwise regression for changes in beta connectivity as they correlate with changes in Fluency.

|  | *β* | *SE β* | *t*(23) | *p*(β) | *F* | *p*(F) | *R^2^* (adj.) |
| --- | --- | --- | --- | --- | --- | --- | --- |
|  |  |  |  |  | 9.56 | < 0.001 | 0.51 |
| Intercept | 0.00 | 0.14 | 0.00 | 1.00 |  |  |  |
| PC1 | -0.04 | 0.02 | -2.21 | 0.04 |  |  |  |
| PC3 | 0.07 | 0.02 | 3.90 | < 0.01 |  |  |  |
| PC6 | 0.04 | 0.02 | 2.15 | 0.04 |  |  |  |

**Table ST4b**

Top 30 edges contributing to the mode correlating Fluency changes based on changes in beta connectivity.

| Edges involved in mode | | Association strength |
| --- | --- | --- |
| Temporal_Sup_R | Supp_Motor_Area_L | -0.56 |
| Caudate_L | Rectus_R | -0.56 |
| Lingual_R | Frontal_Mid_R | -0.55 |
| Caudate_L | Frontal_Med_Orb_R | -0.55 |
| Temporal_Mid_R | Supp_Motor_Area_L | -0.54 |
| Temporal_Mid_R | Cingulum_Mid_L | -0.54 |
| Caudate_L | Frontal_Sup_Orb_R | -0.54 |
| Temporal_Pole_Sup_L | Frontal_Sup_Medial_L | -0.52 |
| Putamen_L | Rectus_R | -0.50 |
| Pallidum_R | Cingulum_Mid_L | -0.50 |
| Temporal_Sup_R | Cingulum_Mid_L | -0.49 |
| Thalamus_R | Insula_L | 0.49 |
| Cuneus_R | Amygdala_L | 0.50 |
| SupraMarginal_L | Lingual_L | 0.50 |
| Temporal_Pole_Sup_L | Parietal_Sup_R | 0.50 |
| Temporal_Pole_Mid_L | Parietal_Sup_R | 0.51 |
| Frontal_Med_Orb_L | Frontal_Mid_Orb_R | 0.51 |
| Precuneus_R | Hippocampus_L | 0.51 |
| Heschl_R | Frontal_Inf_Tri_L | 0.51 |
| Precuneus_R | Insula_L | 0.51 |
| Frontal_Inf_Orb_R | Frontal_Mid_Orb_L | 0.52 |
| Parietal_Sup_R | Amygdala_L | 0.53 |
| Frontal_Inf_Orb_R | Frontal_Sup_Orb_L | 0.54 |
| Parietal_Sup_R | ParaHippocampal_L | 0.55 |
| SupraMarginal_L | Cuneus_L | 0.55 |
| Precuneus_R | ParaHippocampal_L | 0.56 |
| Frontal_Mid_Orb_R | Frontal_Sup_Orb_L | 0.57 |
| Rectus_L | Frontal_Mid_Orb_R | 0.57 |
| Precuneus_R | Amygdala_L | 0.57 |
| SupraMarginal_L | Calcarine_L | 0.58 |
